# The Impact of Indian Teachers of Psychiatry (IToP) Scholarship for Teachers Towards Enrichment of Psychiatry Teaching Skills (STEPS): A Model for Capacity Building of Psychiatry Teachers

**DOI:** 10.1192/bjo.2025.10286

**Published:** 2025-06-20

**Authors:** Kishor Manohar Rao, Kiran Kumar Kadarappa, Vinay Hosagavi Ramalingaiah

**Affiliations:** 1 JSS Medical College, JSSAHER, Mysuru, India; 2 Vydehi Institute of Medical Sciences, RGUHS, Bengaluru, India; 3 Adichuchanagiri Institute of Medical Sciences, ACU, Mandya, India

## Abstract

**Aims:** To assess the impact of Indian teachers of psychiatry (IToP) scholarship for teachers towards enrichment of psychiatry teaching skills (STEPS).

**Methods:** It is a cross-sectional study with Institutional ethical clearance that was carried out online through Google forms from March to Nov 2024. Data was collected through a specifically developed structured questionnaire. Most questions were open-ended questions, and a few were graded on a five-point Likert scale (1 to 5).

**Results:** A total of 16 participants, who were trained from the year 2020 to 2024 in IToP STEPS programme were included in the study. 10 were men and 6 women. Faculty ranged from Professors to Assistant Professors. 75% were involved in teaching both undergraduates and postgraduates. All rated the training as 4/5 on quality that fulfilled the expectation. They felt better equipped with teaching methods, innovative strategies and assessments.

**Conclusion:** Capacity building of psychiatry teachers is much needed to enhance quality of training and thereby psychiatry services. IToP STEPS is a model that can be replicated in other countries where psychiatry education and services is a challenge.

